# Small- bowel mucosal changes and antibody responses after low- and moderate-dose gluten challenge in celiac disease

**DOI:** 10.1186/1471-230X-11-129

**Published:** 2011-11-24

**Authors:** Marja-Leena Lähdeaho, Markku Mäki, Kaija Laurila, Heini Huhtala, Katri Kaukinen

**Affiliations:** 1 Paediatric Research Centre and School of Medicine, University of Tampere and Tampere University Hospital, Tampere, Finland; 2 School of Health Sciences, University of Tampere, Finland; 3 Department of Gastroenterology and Alimentary Tract Surgery, Tampere University Hospital and School of Medicine, University of Tampere, Tampere, Finland

## Abstract

**Background:**

Due to the restrictive nature of a gluten-free diet, celiac patients are looking for alternative therapies. While drug-development programs include gluten challenges, knowledge regarding the duration of gluten challenge and gluten dosage is insufficient.

We challenged adult celiac patients with gluten with a view to assessing the amount needed to cause some small-bowel mucosal deterioration.

**Methods:**

Twenty-five celiac disease adults were challenged with low (1-3 g) or moderate (3-5g) doses of gluten daily for 12 weeks. Symptoms, small-bowel morphology, densities of CD3+ intraepithelial lymphocytes (IELs) and celiac serology were determined.

**Results:**

Both moderate and low amounts of gluten induced small-bowel morphological damage in 67% of celiac patients. Moderate gluten doses also triggered mucosal inflammation and more gastrointestinal symptoms leading to premature withdrawals in seven cases. In 22% of those who developed significant small- intestinal damage, symptoms remained absent. Celiac antibodies seroconverted in 43% of the patients.

**Conclusions:**

Low amounts of gluten can also cause significant mucosal deterioration in the majority of the patients. As there are always some celiac disease patients who will not respond within these conditions, sample sizes must be sufficiently large to attain to statistical power in analysis.

## Background

Celiac disease is an autoimmune-like systemic disorder in genetically susceptible individuals, perpetuated by daily ingested gluten cereals wheat, rye and barley and with manifestations in the small- intestinal mucosa and in organs outside the gut. The gold standard for celiac disease diagnosis is the finding of gluten-induced small-intestinal mucosal injury [1,2]. The mucosa will heal upon introduction of a gluten-free diet and the mucosal damage will reappear if gluten is reintroduced [2]. Extensive time-course studies have provided evidence that during gluten challenge an inflammatory process with a dose-dependent accumulation of intraepithelial lymphocytes (IELs) is followed by mucosal villous atrophy with crypt hyperplasia [3]. In untreated celiac disease the degree of malabsorption is determined by the length of the functionally impaired bowel and the presence of symptoms is noway related to the histological features of a proximal biopsy [3-5]. More recently, it was concluded that only patients with extensive and severe enteropathy will give evidence of steatorrhea and increased intestinal permeability[6]. Biopsy has thus remained the golden standard in measuring gluten-induced ill health.

Previously a gluten challenge was part of the routine diagnostic procedure in children and a characteristic mucosal lesion reappeared in most patients within two years[2,7]. In gluten challenge studies, the amount of gluten in normal food has been estimated to be as high as 10-20 g per day[8-10]. A reduced daily gluten dose (2.5-5 g per day) during a gluten-free diet has still enabled mucosal healing in half of the patients [11]. Also some adults challenged with 2.5-5 g of gluten daily for three to 14 months have shown no significant changes in mucosal morphology [12]. These findings are in marked conflict with those from a recent challenge study where it was shown that even a minimal dose of 50 mg gluten daily induced a significant mucosal deterioration [13]. Altogether, the patient groups in these studies have been rather small and no unequivocal conclusions can be drawn.

A gluten-free diet is socially troublesome and expensive and research has shown compliance to the diet often to be poor [14]. Gluten contamination in a gluten-free diet is also difficult to avoid [15,16]. For these reasons many celiac patients have expressed a desire for other alternative or complementary treatments, less burdensome than a strict life-long gluten-free diet [17,18]. Any drug under evaluation as clinically effective and trusted by celiac disease patients should significantly reduce or prevent gluten-induced mucosal injury. However, data are insufficient as to the amount of gluten to be used in a challenge study and for how long.

In this pilot study we challenged adult celiac disease patients with gluten aiming to gain a clearer conception of the gluten amount needed to cause some mucosal deterioration but without inducing excessive ill health with a resultant dropout of trial subjects. We challenged treated celiac disease patients with low to moderate amounts of gluten for up to 12 weeks to establish whether a morphometrically measurable mucosal deterioration could be detected. We especially measured separately mucosal morphological change as villous height crypt depth ratio (Vh/CrD) and the inflammatory aspect in terms of density of mucosal IELs, both known to be sensitive continuous functional parameters of gluten ingestion in celiac disease. Further, we sought to establish whether the gluten-dependent serum antibody markers show seroconversions.

## Methods

### Patients and study design

Altogether 25 adult volunteers with previously diagnosed celiac disease were recruited for the gluten challenge study. Inclusion criteria comprised biopsy-proven celiac disease in patients aged 18-75 years adhering to a strict gluten-free diet for at least two years and being in clinical remission as judged by an interview, clinical examination and on-site rapid celiac autoantibody testing. Patients with a history of regular dietary transgressions, and those suffering from a condition carrying a risk at endoscopy, or using immunosuppressive medication were excluded.

At the outset the symptoms or signs leading to the diagnosis of celiac disease, the duration of the gluten-free diet, family history of celiac disease and associated conditions were recorded. Upper gastrointestinal endoscopy with duodenal biopsies, laboratory analysis and clinical evaluation including gastrointestinal symptoms, were carried out at baseline and after the challenge. The complete challenge lasted 12 weeks (84 ± 14 days). Furthermore, at 4 weeks (28 ± 7 days) and 8 weeks (56 ± 14 days) the clinical and serological assessments were repeated. The study patients were divided into two groups, those consuming a moderate (3-5 g) amount of gluten and those consuming a low (1-3 g) amount of gluten daily. Those who prematurely withdrew from the study were encouraged to undergo follow-up examinations immediately after the challenge was discontinued. After the challenge all patients continued their normal gluten-free diet.

### Gluten product and dietary assessment

Each patient consumed 1.25-5 g of gluten (as a biscuit) daily during the challenge while otherwise continuing on a strict gluten-free diet. A verified amount of industrial gluten (Meelunie, Amsterdam, the Netherlands) was added to the baking mixture of the biscuits, and the mean daily gluten consumption of each patient was calculated. Consumption of the gluten product, changes in dietary habits and possible dietary transgressions were inquired at each visit and by regular telephone contacts. At baseline and at the end of the study a dietitian made a detailed dietary analysis by means of interview and a 4-day record of food intake.

### Small-bowel mucosal morphology and inflammation

Up to seven small-bowel biopsy specimens were taken from the distal part of the duodenum upon upper gastrointestinal endoscopy; the specimens were evaluated by the same investigator without prior knowledge of the history or findings. At least three biopsies were processed and stained with hematoxylin-eosin and studied under light microscopy. Poorly oriented sections were discarded; when necessary, the samples were dissected repeatedly until they were of good quality. Morphometric analysis measuring Vh/CrD was made in well-oriented biopsy samples as previously described [19], and a ratio < 2.0 was regarded as compatible with villous atrophy and crypt hyperplasia and indicative of active celiac disease. During the gluten-challenge, a decrease in Vh/CrD of 0.5 or more was considered significant and equivalent to clinical glutensensitivity [20].

The remaining biopsies were snap-frozen for immunohistochemical stainings. The specimens were freshly embedded in optimal temperature compound (OCT, Tissue-Tec, Miles Inc, Elkhart, IN, USA), snap-frozen in liquid nitrogen and stored at -70°C. Immunohistochemical studies were carried out on 5-μm-thick frozen sections. CD3+ IELs were stained with monoclonal antibody Leu-4 (Becton Dickinson, San Jose, CA, USA). IELs were counted with a x100 flat-field light microscope objective in randomly selected surface epithelium and the density of IELs expressed as cells/millimetre of epithelium as previously described, the reference values being set at 37 cells/mm for CD3+ IELs [21]. After the gluten challenge an over 30% increase in IEL counts was considered significant and equivalent to clinical glutensensitivity [20]. In our laboratory the correlation coefficients for intraobserver variation for CD3+ IELs has been 0.95 and that for interobserver variation 0.92.

### Celiac serology

Serum IgA class transglutaminase 2 (TG2)-antibodies were investigated by an enzyme-linked immunosorbent assay (ELISA) according to manufacturer's instructions (QUANTA Lite h-tTGIgA, INOVA, Diagnostics, Inc, CA, USA). The cut-off value for a positive antibody level was 20 IU. Serum IgA class endomysial antibodies (EMA) were determined by an indirect immunofluorescence method using human umbilical cord as substrate; a dilution of 1:5 or more was considered positive [22]. Positive sera were further diluted: 1:50, 1:100, 1:200, 1:500, 1:1000, 1:2000, 1:4000. We also determined a combination of serum IgA and IgG class deaminated gliadin peptide (DGP) antibodies (QUANTA Lite Celiac DPG Screen, INOVA Diagnostics) by ELISA, and values ≥20 IU were considered positive. None of the study subjects suffered from selective IgA deficiency. The change in antibody levels was considered clinically significant when antibodies seroconverted from negative to positive or when positive antibody levels increased and at least doubled during the gluten challenge.

### Small-bowel mucosal TG2-targeted autoantibody deposits

The small bowel mucosal TG2- specific autoantibody deposits were studied by direct immunofluorescence methods in frozen biopsy sections [20,23-25]. From each patient altogether six unfixed, 5-μm-thick sections from frozen small-bowel specimens were processed, three for investigation of IgA deposits and three for double-color labelling for both IgA and TG2. IgA was detected by direct immunofluorescence using fluorescein isothiocyanate-labelled rabbit antibody against human IgA (Dako AS, Glostrup, Denmark) as previously described [23]. In celiac disease a clear subepithelial IgA deposition was found below the basement membrane along the villous and crypt epithelium and around mucosal vessels, in contrast to that observed in non-celiac samples, where only endogenous IgA is found inside plasma cells and epithelial cells. Sections were further double-stained for human IgA (green, as above) and for TG2 (red, monoclonal mouse antibody CUB7402, NeoMArers, Fremont, CA, USA, followed by rhodamine-conjugated anti-mouse immunoglobulin antibodies, Dako) to confirm that celiac-type IgA deposits co-localized with TG2. The occurrence of IgA deposits was graded semi-quantitatively according to their intensity along the basement membrane in the villous-crypt area as follows: negative (0), weak (+), moderate (++) and strong positive (+++). In cases where the intensity of the staining was patchy, it was graded from different areas and the mean value was given. All evaluations were carried out in blinded fashion without knowledge of disease history or laboratory findings. The correlation coefficients for both intraobserver and interobserver variations for the detection of presence or absence of TG2-specific IgA deposits have been 0.98 in our laboratory. An increase in the intensity of IgA-deposit staining after gluten challenge was considered indicative of clinical gluten-sensitivity.

### Gastrointestinal symptoms

Clinical symptoms appearing during the gluten challenge were semiquantatively classified into four categories- no symptoms, slight, moderate or severe symptoms- by means of interview and recorded at each visit.

### Statistics

Quantitative data were expressed as medians and ranges or means and 95% confidence intervals (CI). Statistical differences were evaluated using Mann-Whitney test and Wilcoxon's test or unpaired and paired t-test as appropriate, and all testing was two-sided. Fisher's exact test was used in cross-tabulations. The Pearson coefficient was used for correlation studies. P-values < 0.05 were considered statistically significant. All statistical testing was performed using SPSS version 16.0 (SPSS Inc., Chicago, IL, USA)

### Ethical considerations

The study protocol was approved by the Ethics Committee of Tampere University Hospital. All subjects gave written informed consent.

## Results

Altogether 25 adult celiac disease patients fulfilling the inclusion criteria were enrolled for the study (Table 1). At the time of the diagnosis, the majority (88%) had suffered from abdominal symptoms. When volunteering for this study, the patients had been a median of 11 years on a strict gluten-free diet, and all were in clinical remission.

**Table 1:** Demographic data on the 25 celiac disease patients

Variable	
Female; n	21 (81%)
Age; median (range), years	49 (21-68)
Reason for suspicion of celiac disease at diagnosis	
Abdominal symptoms	23 (88%)
Anemia or malabsorption	16 (62%)
Extraintestinal manifestations*	9 (35%)
Screening in risk groups of celiac disease†	1 (4%)
Duration of gluten free diet; median (range), years	11 (2-34)
Family history of celiac disease, n	8 (31%)

*tooth enamel hypoplasia, hypothyreosis, growth delay

† family history of celiac disease

At baseline in the current study, all patients except one had normal Vh/CrD ratios (median 2.9, range 1.3-4.2) on small-intestinal biopsy (Table 2). The mean density of CD3+ IELs was 63 cells/mm (range 23-146). Altogether six patients had borderline positive celiac disease antibody levels (three had positive TG2 antibodies, two EMA and one DGP antibodies); in the rest all three antibodies were negative. When TG2-targeted autoantibodies were assessed at small-bowel mucosal level, moderate IgA-deposits were seen in two and faint in four patients. In the remaining 19 cases, mucosal TG2-autoantibody depositions were negative at the beginning of the challenge study.

**Table 2:** Effect of moderate (3-5g) and low (1-3g) amounts of gluten on small-bowel mucosal villous height crypt depth ratio (Vh/CrD), densities of CD3+ intraepithelial lymphocytes (IELs) and gastrointestinal symptoms in treated celiac disease patients.

No	Mean daily gluten intake (g)	Duration of gluten challenge (days)	Small-bowel biopsy				Symptoms‡	
			Vh/CrD*		CD3+ IELs†			
					
			I	II	I	II	I	II
*Moderate amounts of gluten daily*								
1	5.0	29	2.8	**0.8**	33	**88**	0	**++**
2	4.9	84	3.5	3.4	43	**84**	0	0
3	4.9	38	2.9	**1.3**	64	**128**	0	**++**
4	4.7	45	2.7	**0.2**	70	**113**	0	**+**
5	4.1	61	3.0	3.5	41	**56**	0	**++**
6	4.0	91	3.0	**0.6**	45	**97**	0	**+**
7	3.6	91	2.8	**1.4**	42	**125**	0	0
8	3.6	84	3.0	2.6	38	47	0	**+**
9	3.4	88	3.8	**2.3**	69	83	0	**+**
10	3.3	86	2.7	**0.6**	68	**96**	0	**+**
								
*Low amounts of gluten daily*								
11	2.8	89	2.5	3.1	87	52	0	**+**
12	2.7	84	3.0	**0.6**	87	**117**	0	0
13	2.6	81	2.9	3.1	49	50	0	0
14	2.4	85	2.7	**1.9**	146	**271**	0	**+**
15	2.2	103	4.2	**1.3**	23	**48**	0	**+**
16	2.1	93	1.3	**0.1**	130	99	0	**+**
17	2.1	85	3.3	**2.4**	100	79	0	**+**
18	2.1	84	2.9	**1.7**	75	78	0	0
19	2.1	83	3.2	3.4	70	**123**	0	**+**
20	1.4	77	3.4	3.0	41	**87**	0	**++**
21	1.3	78	2.5	**0.8**	53	**71**	0	0
								
Altogether positive outcome§, n (%)			14 (67%)		14 (67%)		15 (71%)	

Clinically significant changes between baseline (I) and 12 weeks' follow-up examination (II) are marked in bold numbers. Complete challenge lasted 12 weeks (84 ± 14 days).

* Abnormal ratio compatible with active celiac disease <2.0

† Reference value for CD3+ IELs 37 cell/mm

‡ Symptoms graded as 0 = no symptoms, + = mild symptoms, ++ = moderate symptoms,

§ Positive outcome reported when clinically significant changes appeared (>0.5 decrease in Vh/CrD, >30% increase in IEL densities) or when symptoms worsened.

Altogether four celiac disease patients belonging to the moderate gluten dose group discontinued the study due to abdominal symptoms shortly after the challenge was initiated (after three, eight, ten and 23 days), and they were excluded from the final analyses. Celiac serology remained negative in all. The first two drop-outs with extremely brief gluten challenges underwent the follow-up endoscopy, and no small- bowel mucosal deterioration was evident.

The remaining 21 patients comprised the final study population (Tables 2 and 3), for whom the gluten challenge lasted a median of 84 days (range 29-103 days) with an average of 3.1 g daily gluten consumption (range 1.3-5.0 g/day). When the cohort was further divided according to the average daily gluten intake, ten patients belonged to the moderate-dose (3-5 g) and 11 to the low-dose (1-3 g) gluten challenge group (Tables 2 and 3). In the moderate-dose group, three patients withdrew prematurely (after 29, 38 and 61 days) by reason of abdominal symptoms such as diarrhea, vomiting and abdominal pain; one patient discontinued at day 45 for personal reasons. Intended end-point examinations were carried out in all four cases immediately after cessation of the challenge. All celiac patients in the low-dose gluten challenge group completed the study as planned after 12 weeks' challenge. Patients with a moderate gluten dose were thus more prone than those with a low dose to withdraw prematurely from the study (p = 0.035).

**Table 3:** Effect of moderate (3-5g) and low (1-3g) amounts of gluten on serum transglutaminase 2 (TG2) antibodies and small bowel mucosal TG2-targeted autoantibody deposits in treated celiac disease patients.

No	Mean daily gluten intake	Duration of gluten	Serum TG2 antibodies*		Mucosal TG2 antibody deposits†	
			I	II	I	II
*Moderate amounts of gluten daily*						
1	5.0	29	10	**319**	0	**+++**
2	4.9	84	9	13	0	0
3	4.9	38	5	10	0	**+**
4	4.7	45	44	**218**	+	**+++**
5	4.1	61	6	5	0	0
6	4.0	91	21	**263**	+	**+++**
7	3.6	91	6	**47**	0	**++**
8	3.6	84	8	10	++	+
9	3.4	88	13	16	0	0
10	3.3	86	18	**229**	+	**+++**
						
*Low amounts of gluten daily*						
11	2.8	89	6	7	0	**++**
12	2.7	84	7	**111**	0	**++**
13	2.6	81	7	5	0	0
14	2.4	85	12	**88**	0	**++**
15	2.2	103	9	9	0	0
16	2.1	93	16	**94**	++	++
17	2.1	85	9	8	0	0
18	2.1	84	24	21	+	+
19	2.1	83	8	8	0	0
20	1.4	77	10	13	0	**+**
21	1.3	78	14	**101**	0	**++**
						
Altogether positive outcome‡, n (%)			9 (43%)		11 (52%)	

Clinically significant changes between baseline (I) and 12 weeks' follow-up examination (II) are marked in bold letters. Complete challenge lasted 12 weeks (84 ± 14 days).

* Values of ≥20 IU considered positive

† Graded as negative (0), weak (+), moderate (++) and strong positive (+++)

‡ Positive outcome reported when serum antibody levels or intensity of mucosal autoantibody deposits increased.

**Figure 1:**
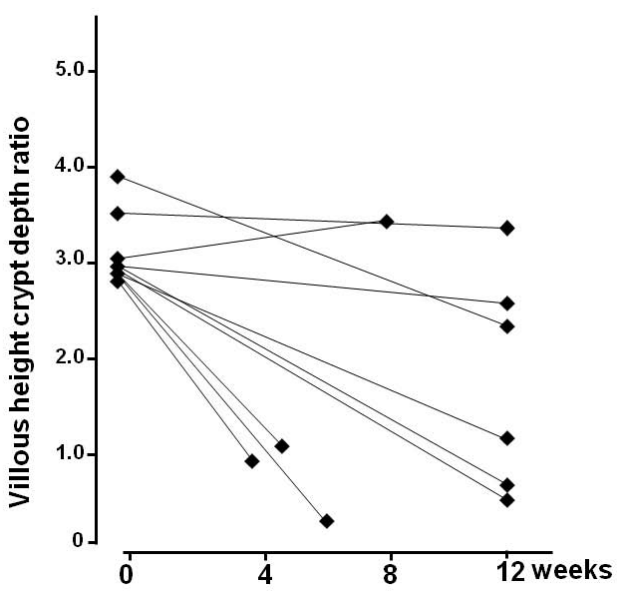
**Changes in small bowel mucosal **v**illous height and crypt depth ratios in moderate-dose group at baseline and at completion of the study**.

**Figure 2:**
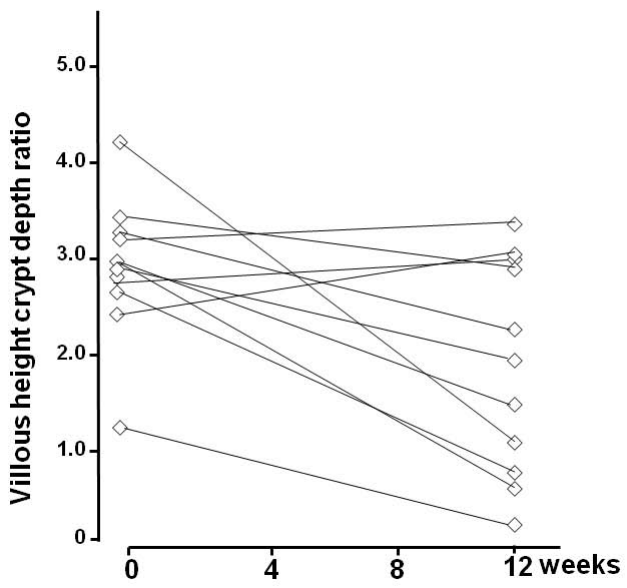
**Changes in small bowel mucosal **v**illous height and crypt depth ratios in low-dose group at baseline and at completion of the study**.

During the gluten challenge, a clinically significant decrease in Vh/CrD was found in 14 (67%) out of the 21 celiac patients; seven were in the moderate and seven in the low gluten dose group (Table 2, Figures 1 and 2). The Vh/CrD in the moderate-dose group deteriorated from a mean 3.0 (95%CI 2.8-3.2) to 1.7 (95%CI 1.0-2.4) and in the low-dose group from 2.9 (95%CI 2.5-3.4) to 1.9 (95%CI 1.2-2.6), both changes being statistically significant (p = 0.002 and p = 0.016, respectively). The changes in Vh/CrD did not correlate with daily gluten intake (r = -0.138, p = 0.552).

**Figure 3:**
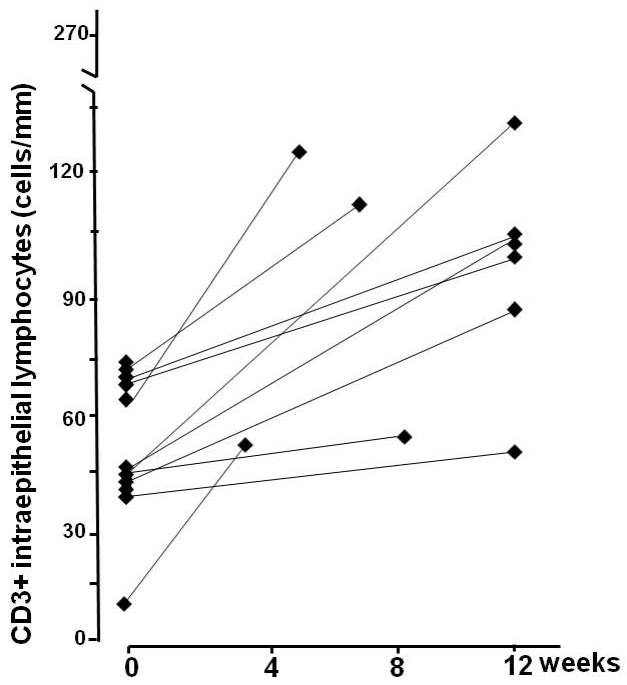
**Changes in small bowel mucosal densities of CD3+ IELs in moderate-dose group at baseline and at completion of the study**.

**Figure 4:**
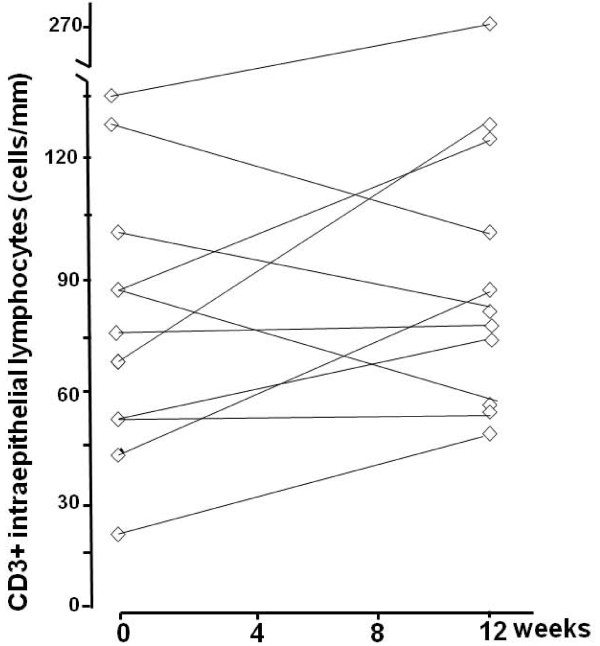
**Changes in small bowel mucosal densities of CD3+ IELs in low-dose group at baseline and at completion of the study**.

In line with the morphological findings, a clinically significant change in the density of CD3+ IELs was seen in altogether 14 (67%) subjects; eight in the moderate-dose and six in the low-dose of (Table 2, Figures 3 and 4). In the moderate gluten dose group the IEL count increased significantly from a mean 49 cell/mm (95%CI 38-60) to 88 cell/mm (95%CI 70-106, p = 0.001). In the low-dose group the increase did not reach statistical significance (from a mean 78 cell/mm [95%CI 56-100] to 98 cell/mm [95%CI 61-135], p = 0.188). All in all, daily gluten intake did not correlate with increased inflammation (gluten intake vs. ΔCD3+ IEL count, r = 0.251, p = 0.273).

**Figure 5:**
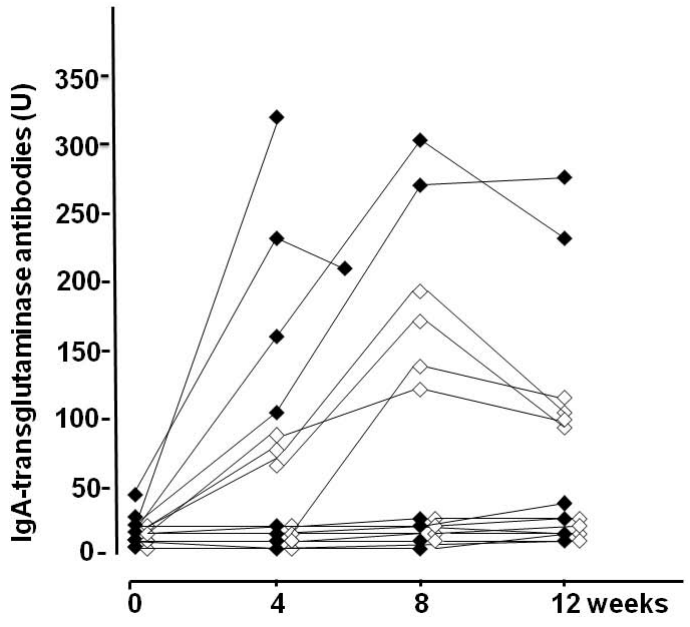
**Changes in antibody levels**. Transglutaminase 2 (TG2) antibody levels at baseline, at 4 and 8 weeks, and at the end of the study. Closed squares indicate moderate daily gluten dose 3-5 g and open squares low gluten doses (1-3 g per day).

Altogether nine (43%) celiac disease patients, five in the moderate- and four in the low-dose gluten challenge group, showed significant increases in TG2 antibody titers (Table 3). The subjects consuming moderate doses of gluten evinced clearly higher TG2 antibody titer responses than those consuming low doses; this was already seen after four weeks' challenge (Figure 5). Interestingly, after the highest positive titer during the gluten challenge, the serum TG2 antibody levels tended to decrease even if gluten consumption continued (Figure 5). Serum EMA and DGP antibody titers were in line with TG2 antibody results; furthermore, after the gluten challenge all these three antibodies appeared in the serum simultaneously (data not shown). Small-bowel mucosal TG2-targeted autoantibody deposits generally followed the gluten-induced serological response in sera and morphological changes seen in the biopsy specimens (Tables 2 and 3).

At the outset, all subjects were free of gastrointestinal symptoms. Altogether 15 (71%) out of the 21 celiac disease patients experienced mild to moderate abdominal symptoms upon gluten challenge; eight of them (including the four withdrawing prematurely, see above) were from the moderate-dose and seven from the low-dose group (Table 2). None developed severe symptoms. Thirteen (87%) out of the 15 symptomatic patients also developed small-intestinal mucosal changes (significant decrease in Vh/CrD and/or increase in the density of CD3+ IELs). Of note, symptoms did not reveal four celiac disease patients having clinically significant gluten-induced small-bowel mucosal damage.

## Discussion

Twelve weeks' gluten challenge with both moderate and low amounts of gluten sufficed to induce small-bowel mucosal morphological damage in the majority of volunteer treated celiac disease adults not, however, in all. An increase in the density of CD3+ IELs paralleled this phenomenon especially in the moderate-dose gluten challenge group. At the same time, moderate gluten doses triggered more gastrointestinal symptoms leading to premature withdrawals from the study. This suggests that in the 12-week time-frame smaller gluten doses of 1-3 g per day might be preferable to higher doses in order to obtain sufficient complete read-outs at the end of a challenge study.

In this study, small-bowel mucosal Vh/CrD measurement seemed to be the most sensitive parameter in both moderate and low gluten doses in assessing gluten-induced damage in celiac disease. This conception is also in line with previous findings, where the Vh/CrD index has been used to show harmful effects of gluten in both short-term gluten challenges and long-term follow-up studies [8,13,26,27]. It is not surprising that the IEL count was a reliable marker for gluten toxicity only with moderate, but not with low gluten doses, as it has previously been shown that in celiac disease during a gluten-challenge, IELs accumulate in the surface and crypt epithelium in a dose-dependent manner [3]. It is also important to note that even if the majority of celiac disease patients after gluten challenge yielded positive read-outs both in the Vh/CrD index and in the IEL count, there were some discrepant cases responding only with one or other parameter (Table 2). Marsh classification entailing six diagnostic categories (type 0-type 3c) has been widely used in the diagnostic work-up and follow-up of celiac disease [3]. Marsh grades type 1-type 3c all indicate cursorily that small-bowel mucosal IEL density is increased (>40 IELs/100enterocytes), but different categories point more specifically to different morphological features (such as normal villous morphology, crypt hyperplasia or villous atrophy). Nonetheless the Marsh classification does not take into account minor gluten- induced mucosal changes (e.g. a significant decrease in Vh/CrD or increase in density of IELs within one Marsh grade) or discrepant results in Vh/CrD or IEL counts during a gluten challenge. It would thus be advisable in gluten challenge research projects, to use two separate continuous readout parameters in assessing gluten-dependent functional mucosal changes, i.e. the morphological Vh/CrD and the inflammatory IEL changes, as opposed to applying a simplified grouped classification.

Even if abdominal symptoms after the consumption of cereals do not generally constitute a specific marker of untreated celiac disease [28], gluten-induced gastrointestinal symptoms in our challenge study showed high positive predictive value (87%) for small-bowel mucosal injury in celiac disease patients. However, in 22% of the cases who developed significant small-intestinal damage, symptoms remained absent. It is widely known that many untreated celiac disease patients do not suffer from symptoms even if they have a manifest gluten-induced small-bowel mucosal lesion [29]. Furthermore, in some treated celiac disease patients, symptoms may disappear on a strict gluten-free diet despite continued ongoing mucosal villous atrophy [30,31]. Such persistent small-intestinal villous atrophy in symptom-free patients clearly carries a risk of subsequent severe complications such as osteoporosis and malignancies [30]. This warrants caution in relying solely on symptoms or other patient-related outcomes without small-bowel biopsies in gluten challenge research projects.

In studies where a gluten challenge has been applied for diagnostic purposes in children on a normal daily gluten-containing diet (10-20 g daily), a small-bowel mucosal histological relapse has usually developed within three to twelve months in the majority of patients [8,9]. Seroconversion of EMA and TG2 antibodies is known to be a reliable predictor of histological relapse and antibody testing has helped in the timing of the biopsy [9]. In this study with low to moderate gluten doses, only 43% of the challenged adult celiac disease patients evinced a significant serological response. In line with earlier challenge studies, here all those who had increased serum autoantibodies during the challenge also developed small-bowel villous atrophy. However, at the same time serology missed nine cases with significant small-bowel mucosal changes (Tables 2 and 3). These cases might have called for a longer gluten challenge period or higher gluten amounts. However, it is well-known that serology may fail to detect occasional slight dietary transgressions and incomplete mucosal healing [30,32,33], and therefore celiac serology is not always a reliable means of assessing gluten-induced harm. Furthermore, it is important to note that after the gluten challenge all three antibody - EMA, TG2 and DPG antibody - tests performed similar way, and recently introduced DPG antibody test was not superior to conventional serological assays. As small-bowel mucosal TG2-specific autoantibody deposits have also been found in seronegative celiac patients and patients with early-stage celiac disease [23,24,34], it has been hypothesized that these mucosal autoantibodies might also appear early during a gluten challenge. In our study, mucosal autoantibodies followed the morphological damage, and they added only little to the normal morphometrical measurements. It might be that after a short-term acute gluten challenge the distribution of mucosal autoantibody depositions is patchy, and single mucosal biopsies may not reveal the positivity. It remains to be seen whether they will be of use at earlier time-points in the gluten challenge, but due to the invasive nature of small-bowel biopsies we were not able to take specimens on several occasions during the challenge.

There were some celiac disease patients who did not respond in terms of any of the measured outcome variables during the 12 weeks' gluten challenge. It is well-known that celiac patients show varying sensitivity to gluten and the process of mucosal deterioration may take years or even decades [35-37]. Furthermore, it should be remembered that the phenotype of the disease may vary in the same person over time; celiac patients suffering originally from abdominal symptoms at diagnosis may later experience only extraintestinal symptoms such as dermatitis herpetiformis when gluten is re-introduced into the diet [37]. These issues need to be taken into account when sample sizes in study cohorts are determined.

At the baseline of the study, the majority of the treated celiac disease patients evinced increased densities of IELs and one patient even had villous atrophy with crypt hyperplasia despite adherent to a strict long-term gluten-free diet. According to the literature, such cases exist and it has been shown that even 80% of treated patients may have ongoing villous atrophy during a strict diet [38-40]. These persistent mucosal changes might be due to constant residual gluten contamination in the food, which again would indicate that alternative treatment options would be desirable.

## Conclusions

All in all, further studies are still needed to establish the optimal gluten dose on the other hand causing some small-bowel mucosal deterioration but without inducing excessive ill health. Our results suggest that in drug trials, the duration of the gluten challenge might be shorter than 12 weeks and the gluten dose could be an average of those used in the current study (approximately 3g). As there are always some celiac disease patients who will not respond within these conditions, sample sizes must be sufficiently large to ensure adequate statistical power for the study. The Vh/CrD index seemed to be the most reliable outcome measurement in both moderate and low gluten doses. Furthermore, it must be remembered that during a gluten challenge, some celiac patients may remain asymptomatic even if they develop manifest gluten-induced mucosal damage - this might in the long-run predispose these cases to celiac disease-related complications.

## Abbreviations

IELs: intraepithelial lymphocytes; Vh/CrD: villous height crypt depth ratio; TG2: transglutaminase 2; EMA: endomysium antibodies; DGP: deaminated gliadin peptide; ELISA: enzymelinked immunosorbent assay; CI: confidence interval.

## Competing interests

The authors declare that they have no competing interests.

## Authors' contributions

MLL participated in the study design, carried out the patient visits and helped to draft the manuscript. MM was involved in study design and preparing the manuscript. KL was involved in laboratory assessments and critically reviewed the manuscript. HH performed the statistical analysis and reviewed the manuscript critically. KK participated in the study design, was involved in the laboratory assessments and drafted the manuscript. All authors read and approved the final manuscript.

## Pre-publication history

The pre-publication history for this paper can be accessed here:

http://www.biomedcentral.com/1471-230X/11/129/prepub
